# Yeast Multi-Enzymatic Systems for Improving Colour Extraction, Technological Parameters and Antioxidant Activity of Wine

**DOI:** 10.17113/ftb.60.04.22.7777

**Published:** 2022-12

**Authors:** Sara Jaquelina Longhi, María Carolina Martín, María Gabriela Merín, Vilma Inés Morata de Ambrosini

**Affiliations:** 1 National University of Cuyo, Faculty of Applied Sciences to Industry, Bernardo de Irigoyen 375, 5600 San Rafael, Mendoza, Argentina; 2 National Scientific and Technical Research Council (CONICET), Ciudad Autónoma de Buenos Aires, Argentina

**Keywords:** antioxidant activity, colour extraction, technological parameters, plant cell wall-depolymerizing enzymes, winemaking, yeast

## Abstract

**Research background:**

Wine yeasts are a heterogeneous microbial group with high enzymatic potential that makes them a useful tool in winemaking. With a better understanding of their oenological properties, selection procedures can be optimised to obtain more efficient strains. The present study aims to isolate and select yeasts from wine grape surface by studying their production of enzymes that hydrolyse plant cell wall polymers and by linking them to different technological parameters and antioxidant activity of wines.

**Experimental approach:**

Yeasts that are able to produce carbohydrolases and related enzymes of oenological importance were firstly selected on plates and subsequently identified. Then, a secondary selection of yeasts was carried out according to technological effects of their extracellular enzyme extracts on short macerations. In this way, the colour extraction, total polyphenol content, clarification, filterability and antioxidant activity were studied. This approach makes it possible to correlate the microorganism capacity to produce cell wall-depolymerizing enzymes with their technological effects.

**Results and conclusions:**

From 366 isolates, 96 strains (26.2%) showed at least one of the polysaccharidase activities and 55 strains (57.3%) of them exhibited activities of multiple enzymes that degrade plant cell wall polymers. Sixteen strains were selected and identified as *Aureobasidium*, *Candida, Debaryomyces*, *Hanseniaspora, Metschnikowia, Pichia, Saccharomyces* and *Torulaspora*. Pectinolytic enzymes had the highest hydrolytic activity. *Aureobasidium pullulans* had a broader enzyme blend and higher activity, dominated by pectinases and followed by xylanases and cellulases. Moreover, the *Torulaspora delbrueckii* m7-2 strain produced high amounts of polysaccharidase and this was strain-dependent. Strains that produced enzyme extracts with a wide range of activities that were also the highest, also had the best chromatic and technological properties. Cluster analysis confirmed that *A. pullulans* R-22, m11-2, m86-1 and m86-2 and *T. delbrueckii* m7-2 could be correlated with a better effect on filterability, clarification and extraction of bioactive compounds, encouraging future studies regarding their application in winemaking.

**Novelty and scientific contribution:**

The study of yeast multi-enzymatic systems impacting the grape maceration process enables a proper selection criterion for wine yeasts to improve colour extraction, technological parameters and antioxidant activity of Malbec wine. This work shows that *A. pullulans* and *T. delbruekii* have a high enzymatic potential for oenological purposes.

## INTRODUCTION

Winemaking is a dynamic microbiological process that involves a number of yeast genera, species and strains. *Saccharomyces cerevisiae* is the species that is responsible for carrying out the alcoholic fermentation. Other yeasts present in the early stages of winemaking are so-called non-*Saccharomyces* species, which provide special characteristics to the final product because of their ability to produce extracellular metabolites and enzymes such as pectinases, cellulases, xylanases, glycosidases, proteases or esterases (*1*–*4*). Some unconventional species in winemaking worth mentioning are epiphytic grape microbiota, which provide novel enzymes of oenological interest that help the wine industry face technical and consumer challenges (*5*).

The colour of red wine is an easily perceived quality attribute that is affected by various viticultural and wine-producing factors. Quality wines usually contain high amounts of non-volatile phenolic compounds, which are important contributors to flavour, colour, mouthfeel (astringency and bitterness) and health promoting properties (*e.g*. antioxidant and anti-inflammatory metabolites) of wine (*6*). The major phenolic compounds found in grapes and wines are hydroxycinnamic acids, stilbenes, anthocyanins and tannins (*7*). Currently there is a great interest in the application of technological strategies that increase the phenolic composition and hence antioxidant activity of musts and wines. Maceration process is crucial for the extraction of these compounds. In particular, cold pre-fermentation macerations carried out at 5–15 °C improve the colour and stability of red wine, and increase the production and retention of aromatic compounds with the consequent enhancement of the flavour of the wines (*8*).

However, low temperatures reduce the extraction of phenolic compounds due to a decrease in molecular mobility. This reduced extraction can be compensated and/or enhanced with the use of cold-active enzyme extracts, as previously demonstrated (*9*–*11*). They consist of a pool of depolymerizing enzymes, mainly pectinases, which play an essential role in the degradation of cell wall polymers, alongside secondary enzyme activities that specifically act on other components of wine must (*12*, *13*).

Pectinolytic enzymes are a heterogeneous group of enzymes that degrade pectin present in the middle lamella and primary cell walls of plants, affecting both the sensory and technological properties of wines (*10*, *14*). Pectinases can help improve clarification and filtration by releasing more colour and flavour compounds contained in the grape skin, and make the release of phenolic compounds more effective (*1*). Cellulases and hemicellulases degrade polysaccharides from the cell walls of the skin and pulp of the grape, a process that contributes to the extraction of juice, improves clarification of the wine and increases the fruity aroma of the wines through the release of aromatic precursors (*15*). Furthermore, glycosidases and proteases are some of the enzymes of oenological interest produced by epiphytic yeasts. The former ones improve the aromatic fraction of wines by releasing aromatic terpenes present in the must in the form of glycosylated precursors. Brandolini *et al*. (*16*) and Gaensly *et al*. (*17*) also showed that yeasts with β-glucosidase activity were able to increase the concentration of free resveratrol in wine and increase its antioxidant capacity. Proteases, for their part, improve the nutritional content of musts, contribute to the production of aromatic precursors, reduce protein content and facilitate the protein stability of the wine (*1*). Enzymes found in natural yeasts on the grape carposphere that are adapted to their ecological environment contribute to the release of essential pigments and compounds to wine. This process would not cause any changes other than those related to the natural course of the process, and would thus increase the typicality of the wines, a trend increasingly valued by consumers.

Regarding the addition of enzymes in the process of making red wine and based on the results of previous studies (*3*, *4*, *9*), the activities of different polysaccharidases and those of related enzymes that constitute enzyme preparations perform different hydrolytic actions on the cell walls and produce complementary effects to efficiently break down cell walls, resulting in an enrichment of the composition of wine must. Recently, scientific advances have been reported regarding the chemical and structural composition of the grape berry cell wall, and the hydrolytic action on its polysaccharide components by commercial enzymes. The results revealed different cell wall compositions in the different cell strata and the need for unique enzyme activities to achieve its complete degradation (*12*, *18*). These findings indicate the need to search for complementary enzyme activities to effectively and completely degrade grape berry cell walls.

In our previous studies (*3*, *4*, *19*, *20*), we reported the pectinolytic yeasts isolated from grape berries and must, and winery equipment; however, these studies mainly focused on cold-active pectinolytic activity and were not based on the pool of carbohydrolases and related enzymes of oenological importance. In the present study, the objective is to select microorganisms from the wine grape surface that produce multi-enzyme systems based on the physicochemical and technological performance during short macerations of Malbec must. Therefore, here we used broader criteria for the selection than those used in previous studies. The pool of hydrolytic activities degrading plant cell wall polymers was assayed at two maceration temperatures, and compared with different technological effects, colour extraction and the antioxidant activity of the resulting macerations.

## MATERIALS AND METHODS

### Study area, sampling and microorganism isolation

Grape samples were taken from the San Rafael wine region, Mendoza, Argentina. Microorganisms were isolated from the surface of grape berries of Bonarda, Cabernet Sauvignon and Malbec cultivars (*Vitis vinífera* L.). Twenty grape berries of each cultivar were taken, placed in a container with 20 mL of sterile 0.1% peptone water and stirred for 1 h at 165 rpm (0.6×*g*). Samples were plated on solid WL (Wallerstein Laboratory) medium, nutrient agar and malt extract agar (MEA). The incubation time was 5 and 7 days at 28 and 15 °C, respectively. Culture media were purchased from Sigma-Aldrich Co. Ltd., Merck KGaA (Darmstadt, Germany).

### Primary selection

Plate screening for the selection of microorganisms producing grape berry cell wall-depolymerizing enzymes

For detection of pectinase activity, isolates were inoculated in mineral medium with citric pectin as the sole carbon source according to the method by Moyo *et al*. (*21*) with modifications and with the following composition (in g/L): citric pectin 2.0, yeast extract 1.0, agar 15.0, KH_2_PO_4_ 0.2, CaCl_2_ 0.05, (NH_4_)_2_SO_4_ 1.0, MgSO_4_·7H_2_O 0.8, MnSO_4_ 0.05; pH=4.5. To demonstrate xylanase, cellulase and amylase activities, isolates were plated onto selective medium containing 6.7 g/L yeast nitrogen base (YNB) and 20 g/L agar, plus 0.2% birchwood xylan for xylanase, 0.5% carboxymethylcellulose for cellulase or 2% starch for amylase activity. All reagents and YNB medium were from Sigma-Aldrich Co. Ltd., Merck KGaA. Enzyme production was detected using the qualitative method that consists of the development of hydrolysis halos. Pectinase, xylanase and amylase were detected with a lugol solution (*22*) and cellulase activity with a 0.2% (*m*/*V*) Congo Red solution. Activity was detected when a clarification halo was formed around the colonies against a brown-purple background of the medium with non-degraded polymers after all polysaccharidases had been developed, except for amylase, which had a blue background (*23*).

#### Plate screening for other hydrolytic activities of oenological importance

Extracellular protease activity was assayed qualitatively by point inoculation of yeasts on plates with skimmed milk agar and gelatine agar (Sigma-Aldrich Co. Ltd., Merck KGaA) at pH=4.5, according to Charoenchai *et al.* (*24*). Skimmed milk agar plates were directly examined for clear zones surrounding yeast growth after incubation, whereas gelatine agar plates were flooded with 10 mL acetic acid (50 g/L) prior to examination of clear zones around the yeast cells.

β-Glucosidase activity was assayed following Villena *et al.* (*25*) in a medium containing 0.5% cellobiose (4-O-β-d-glucopyranosyl-d-glucose; Sigma-Aldrich Co. Ltd., Merck), 0.67% YNB and 2% agar. Prior to incubation, a pure isolated colony was suspended in 1 mL of sterile distilled water in an Eppendorf tube. The yeast suspension was then centrifuged at 2500×*g* for 5 min at 4 °C (Rolco Srl, Buenos Aires, Argentina), the supernatant was discarded and the precipitate washed twice with sterile distilled water to eliminate the remaining nutrients from the initial medium. Finally, the precipitate was suspended in sterile distilled water and seeded with a loop onto the solid medium containing cellobiose. Positive activity was determined by colony growth. *Torulaspora delbrueckii* BTd259 and *Debaryomyces vanrijiae* BDv566 were used as positive control strains for β-glucosidase (*2*, *13*).

### Pheno- and genotype identification of selected yeasts

Yeasts demonstrating highest hydrolytic enzyme production were identified at species level following the taxonomic criteria described by Kurtzman *et al.* (*26*) based on their morphological and physiological characteristics as well as the polymerase chain reaction-restriction fragment length polymorphism (PCR-RFLP) analysis of the ITS1-5.8S–ITS2 region from the rRNA gene. PCR was carried out according to protocols described by Esteve-Zarzoso *et al.* (*27*) with some modifications using universal primers ITS1 (5′-TCCGTAGGTGAACCTGCGG-3′) and ITS4 (5′-TCCTCCGCTTATTGATATGC-3′; Integrated DNA Technologies, Coralville, IA, USA), already described by White *et al.* (*28*), using a thermal cycler TC-312 (Techne, Waltham, MA, USA). PCR products were digested with *Cfo*I, *Hinf*I and *Hae*III restriction enzymes following the supplier's instructions (Promega Co., Madison, WI, USA). Amplified products and their restriction fragments were electrophoresed on 1.4 and 2.2% agarose gels, respectively, in 1×TAE (Tris-acetic acid-EDTA) buffer, using a horizontal electrophoresis cell (Labnet International, Inc., Edison, NJ, USA). Gels were stained with ethidium bromide, visualized and photographed under UV light (NYX Technyk Inc., San Diego, CA, USA). Fragment sizes were estimated by comparison with a DNA standard (100-bp ladder). All PCR-RFLP reagents were purchased from Promega Co.).

### Pectinolytic yeasts used as reference strains

In addition to the microorganisms isolated in the present study, the following pectinolytic microorganisms were obtained from the Biodiversidad San Rafael (Mendoza) culture collection of the FCAI-UNCUYO (San Rafael, Mendoza, Argentina) belonging to the SCCM-AAM (Argentine Association of Microbiology), affiliated to the FELACC (Latin-American Federation of Culture Collections): *Aureobasidium pullulans* GM-R-22 (in this manuscript referred to as Ap-R-22), *Cryptococcus saitoi* GM-4 (Crs-GM-4), *Filobasidium capsuligenum* B-13 (Fc-B-13), *Rhodotorula dairenensis* GM-15 (Rd-GM-15) and *S. cerevisiae* B-17 (Sc-B-17). These strains had been selected in our previous works (*3*, *4*, *19*, *20*) for their pectinolytic activity with oenological importance and were included in the present study to expand the analyses of unexplored aspects.

### Production of extracellular enzyme extracts

The yeasts under study were inoculated in liquid medium according to Moyo *et al.* (*21*) with modifications and with the following composition in 50 mM citric-citrate buffer, pH=3.8 (in g/L): CaCl_2_ 0.05, KH_2_PO_4_ 0.2, MnSO_4_ 0.05, (NH_4_)_2_SO_4_ 1.0, citric pectin 1.0, dextrose 1.0.; Sigma-Aldrich Co. Ltd., Merck KGaA. Cultures were incubated under shaking conditions (130 rpm, *i.e.* 0.4×*g*) at 28 °C for 3 days, using a water bath shaker (SHZ-88; Semedix, Buenos Aires, Argentina). Cells were separated by centrifugation (10 000×*g* for 15 min at 4 °C; Presvac EPF-12R, Buenos Aires, Argentina) and cell-free supernatants were used as enzyme extracts.

### Quantitative evaluation of enzyme activities under oenological conditions

Pectinolytic activity was assayed by measuring the amount of reducing sugars released from a pectin dispersion (0.25% pectin in 50 mM citric acid-citrate buffer, pH=3.8) using 3,5-dinitrosalicylic acid (DNS) reagent (*29*). Galacturonic acid was used as standard. All reagents were purchased from Sigma-Aldrich Co. Ltd., Merck KGaA. Reaction mixtures (enzyme extract/substrate ratio 1:10) were incubated at 15 and 28 °C for 20 min. One pectinase unit (U) was defined as the enzyme activity that released 1 µmol of reducing sugars per min under the given assay conditions.

The conditions for the determination of cellulase, xylanase and amylase activities were the same as those described for pectinolytic activity but using 0.25% (*m*/*V*) carboxymethyl cellulose, 0.25% (*m*/*V*) birchwood xylan and 1% (*m*/*V*) soluble starch (Sigma-Aldrich Co. Ltd., Merck KGaA), dissolved in 50 mM citric acid-citrate buffer (pH=3.8). The reaction mixture was incubated for 20 min at 15 and 28 °C. One unit of cellulase, xylanase or amylase activity was defined as the activity necessary to produce 1 µmol of reducing sugars (glucose or xylose) per min under the assay conditions.

β-Glucosidase activity was assayed by incubating 100 µL of enzyme extract with 100 μL of 15 mM d-(+)-cellobiose (Sigma-Aldrich Co. Ltd., Merck KGaA) solution in 50 mM citric acid-citrate buffer (pH=3.8) at 15 and 28 °C for 30 min. Glucose production was quantified using the enzymatic colorimetric glucose oxidase peroxidase (GOD-POD) method (Wiener Lab, Rosario, Argentina) (*25*). One β-glucosidase unit was defined as the enzyme activity necessary to release 2 µmol of glucose from cellobiose per min under the assay conditions.

### Secondary selection

#### Technological effects of extracellular enzyme extracts on short macerations of grape must

A secondary selection of yeasts according to technological criteria was made by evaluating the effects of extracellular enzyme extracts on short macerations of grape must. Short macerations were carried out in 50-mL Falcon tubes containing 40 g of Malbec grape must and supplemented with 1 mL of the corresponding enzyme extracts (1 U/mL). Control treatments without enzyme (reaction blank) were obtained by replacing the enzyme extracts with 1 mL of citric acid-citrate buffer, and a reference treatment, using a commercial enzyme (Extrazyme, Institut Oenologique de Champagne, Épernay, Marne, France), supplemented at an amount of identical net enzyme units (EU), were also performed. All maceration assays were incubated for 6 h at 15 and 28 °C. All experiments were carried out in triplicate.

#### Colour parameters

Colour extraction was assayed by examining classic vinification parameters. Colour intensity (CI) was determined spectrophotometrically (HACH DR6000; Onelab, Buenos Aires, Argentina) by summarizing the absorbances at 620, 520 and 420 nm of undiluted must, using 1 mm optical path cuvettes, according to Glories (*30*). The hue was calculated as the quotient between the absorbances at 420 and 520 nm according to Sudraud (*31*). CIELAB coordinates (L*, a* and b*) were determined according to the standard method of the Commission Internationale de l'Éclairage (*32*). The CIELAB colour difference (ΔE*) was calculated using the following equation:

ΔE*=[(ΔL*)^2^+(Δa*)^2^+(Δb*)^2^]^1/2^ /1/

where the diﬀerence (Δ) is calculated for each independent variable between the enzymatically treated macerations and its respective controls.

#### Total polyphenol index and total polyphenol content

The total polyphenol index (TPI) was determined with a 100× diluted must sample with distilled water; absorbance was determined at 280 nm according to Glories (*30*) and Ribéreau-Gayon *et al*. (*33*). Finally, total polyphenol content (TPC) was determined according to the classic Folin-Ciocalteu (FC) method (*34*) and expressed in mg gallic acid equivalents (GAE) per L of sample. Briefly, 0.1 mL of must was mixed with 0.1 mL FC reagent (1 mol/L) and incubated for 2 min. Next, the solution was mixed with 0.2 mL of 20% sodium carbonate and 1.6 mL water. The reaction solutions were incubated at room temperature for 25 min and then the absorbance was measured at 730 nm.

#### Clarification and filterability

A volume of 250 mL of enzyme extract was added to 10 mL of Chardonnay white grape must. After enzyme treatment, clarification was evaluated by determining the transmittance (in %) as a measure of clarification at 650 nm, with distilled water as a reference, using a spectrophotometer (Metrolab, Lima, Perú).

After the enzyme treatment described above, the must was vacuum filtered (25 mm diameter filter, 14 µm pore size, 0.9 bar; Sartoruis, Göttingen, Germany) as described by Belda *et al*. (*1*) with modifications. Filterability was expressed in seconds needed to filter 1 mL of clarified must.

#### Antioxidant activity

Antioxidant activity was determined using the 2,2-diphenyl-1-picrylhydrazyl (DPPH˙) radical scavenging method, according to the modified technique by Brand-Williams *et al.* (*35*). The reaction mixture was prepared with 100 µL of sample (1:10 dilution) and 2.9 mL of DPPH˙ solution (0.03 mg/mL), the reaction time was set at 25 min and spectrophotometric readings were performed at 515 nm. All determinations were carried out at room temperature, and the results are expressed as antioxidant capacity in mg of GAE per L of sample. Reagents were purchased from Sigma-Aldrich Co. Ltd., Merck KGaA.

### Statistical analysis

All experimental data are the average of three repetitions±standard deviation. Analysis of variance (ANOVA) was applied to these data, and the mean values were compared by means of Fisher's test of significant differences, with a level of significance of p<0.05, and using Statgraphics Plus software v. 5.1. (*36*). In addition, R software v. x64 3.6.3 (*37*), Rcmdr and FactoMindeR were used for principal component analysis (PCA).

## RESULTS AND DISCUSSION

### Screening of enzyme activities and identification of wine grape yeasts

A total of 366 yeast and yeast-like microorganisms was isolated and 96 of them showed pectinase, xylanase and cellulase activities with the plate technique, indicating that 26.2% of the isolates showed at least some of the assayed enzyme activities. Of the 96 isolates demonstrating enzyme activity, 28 only presented pectinase and 13 only xylanase activity, while no isolate showed cellulase activity without being positive for the other two activities. This suggests that cellulolytic activity would be an adjunct to pectinolytic and xylanolytic activity during degradation of plant organic matter. The remaining isolates had at least two of the assayed activities, indicating that 57.3% of the isolates with enzyme activity showed the activity of multiple enzymes that are necessary to degrade a material as complex as the plant cell wall.

A primary isolate selection was carried out based on the halo diameter *vs* colony diameter ratio (*D*_h_/*D*_c_) at 15 and 28 °C, selecting isolates that presented *D*_h_/*D*_c_ ratios between 2 and 7 and a colony diameter greater than 5 mm. Additionally, other enzyme activities complementary to polysaccharidases such as proteases, amylases and β-glucosidases were assayed for their positive effect on various technological and sensorial parameters. As can be seen in Table 1, nine yeast and yeast-like isolates were selected at 15 °C and 7 at 28 °C.

**Table 1:** Strains selected at 15 and 28 °C based on the halo diameter/colony diameter ratio (*D*_h_/*D*_c_) and substrate degradation, resulting from screening on solid medium

Strain	Pectinase	Xylanase	Cellulase	Amylase	Protease	β-Glucosidase
*D*_h_/*D*_c_			
*t*=15 °C						
m86-2	3.50	2.45	3.91	1.50	+	+
m86-1	2.36	2.00	2.20	1.80	-	+
m89-1	4.00	2.00	2.20	nd	-	-
m50	4.25	3.33	2.22	nd	-	-
m87-3	2.00	4.50	2.80	nd	+	+
m82	3.00	3.50	2.00	nd	-	+
ts-1	3.00	2.88	2.20	nd	+	-
ts-2	3.50	2.29	2.80	nd	+	-
m79-1	3.36	2.09	2.10	nd	-	+
*t*=28 °C						
m11-2	3.80	3.20	4.55	2.10	+	+
m27-2	6.67	6.67	2.75	nd	-	-
m18	4.25	2.88	2.20	nd	+	-
m28-1	2.50	7.00	2.50	nd	+	+
m7-2	4.00	3.38	2.40	nd	-	+
m30-1	2.00	3.50	2.50	nd	-	-
m29	2.30	4.29	2.20	nd	-	-
nd=not detected						

Table 2 shows the identification of the selected strains based on PCR-RFLP analyses of the ITS1-5.8S-ITS2 region of the rRNA gene, defining them as *Aureobasidium* (3 strains), *Candida* (1), *Debaryomyces* (2), *Hanseniaspora* (2), *Metschnikowia* (3), *Pichia* (1), *Saccharomyces* (1) and *Torulaspora* (3) genera. It can be seen that the ability to produce polysaccharidases, necessary to survive in such a complex niche, was present in various microbial genera. This result differs from that reported by Belda *et al.* (*1*), who assayed 462 yeast isolates for different enzyme activities of oenological interest and observed that only the *Metschnikowia* and *Aureobasidium* genera were positive for polygalacturonase activity, while cellulase activity was only observed in *Aureobasidium*.

**Table 2:** Identification of selected strains based on polymerase chain reaction-restriction fragment length polymorphism (PCR-RFLP) analyses of the ITS1-5.8S-ITS2 region of the rRNA gene

Strain	Species^a^	Taxonomy	AP^b^/ bp	Restriction length/bp		
*Cfo*I^c^	*Hae*III^d^	*Hinf*I^e^
m11-2	*Aureobasidium pullulans*	*Euascomycete*	600	190+180+100	450+150	290+180+130
m86-2	*Aureobasidium pullulans*		600	190+180+100	450+150	290+180+130
m86-1	*Aureobasidium pullulans*		600	190+180+100	450+150	290+180+130
m89-1	*Candida stellata*	Oxidative or weakly fermentative *Hemiascomycete*	500	220+130	470	250+230
m50	*Debaryomyces hansenii*		650	320+300+50	420+150+100	330+320
m87-3	*Debaryomyces vanrijiae*		650	310+300+50	420+140+90	320+320
m27-2	*Hanseniaspora* sp.		750	330+320+100	760	330+180+150+80
m82	*Hanseniaspora* sp.		750	320+310+100	740	330+180+150+70
m18	*Metschnikowia pulcherrima*		400	210+100+80	280+100	200+180
ts-1	*Metschnikowia pulcherrima*		400	210+100+80	280+100	200+180
ts-2	*Metschnikowia pulcherrima*		400	210+100+80	280+100	200+180
m28-1	*Pichia guilliermondii*		630	300+260+80	400+110+80	320+300
m79-1	*Saccharomyces cerevisiae*	Fermentative *Hemiascomycete*	880	390+360	320+220+180+150	360+150
m7-2	*Torulaspora delbrueckii*		800	330+220+150+100	800	410+380
m30-1	*Torulaspora delbrueckii*		800	330+220+150+100	800	410+380
m29	*Torulaspora pretoriensis*		825	380+330+110	800	380+290+125
^a^Species assigned according to Esteve-Zarzoso *et al.* (*27*) or using YEAST-ID database (www.yeast-id.org), ^b^AP=amplified product size, ^c,d,e^restriction enzymes used						

According to Barata *et al.* (*38*), the different genera found on the grape surface can be classified into three main groups, which are successively detected as maturation progresses: (*i*) oligotrophic oxidative basidiomycetous yeasts, the yeast-like fungus *A. pullulans* and lactic acid bacteria, (*ii*) copiotrophic oxidative ascomycetes (several *Candida* spp.), weakly fermentative apiculate yeasts (*Hanseniaspora* spp.), film-forming yeasts (*Pichia* spp.) and fermentative yeasts (*C. zemplinina*, *Metschnikowia* spp.), and (*iii*) copiotrophic strong fermentative yeasts (*Saccharomyces* spp., *Torulaspora* spp.). In agreement with this classification and because the samples were ripe grapes, our results revealed a great diversity of strains belonging to the three microbial groups on the grape surface. The ability to produce cell wall-degrading enzymes may be closely related to these population dynamics. Consequently, during the first stages, oligotrophic microorganisms that are able to survive with few available nutrients are more abundant. These microorganisms produce enzymes that make gaps in the plant cell walls, thus creating favourable conditions like a greater availability of nutrients for the development of copiotrophic species. The latter species do not require their own enzyme activities because the enzymes for the degradation of cell walls would already be present in the environment.

### Hydrolytic enzyme activities under oenological conditions

**Fig. 1:**
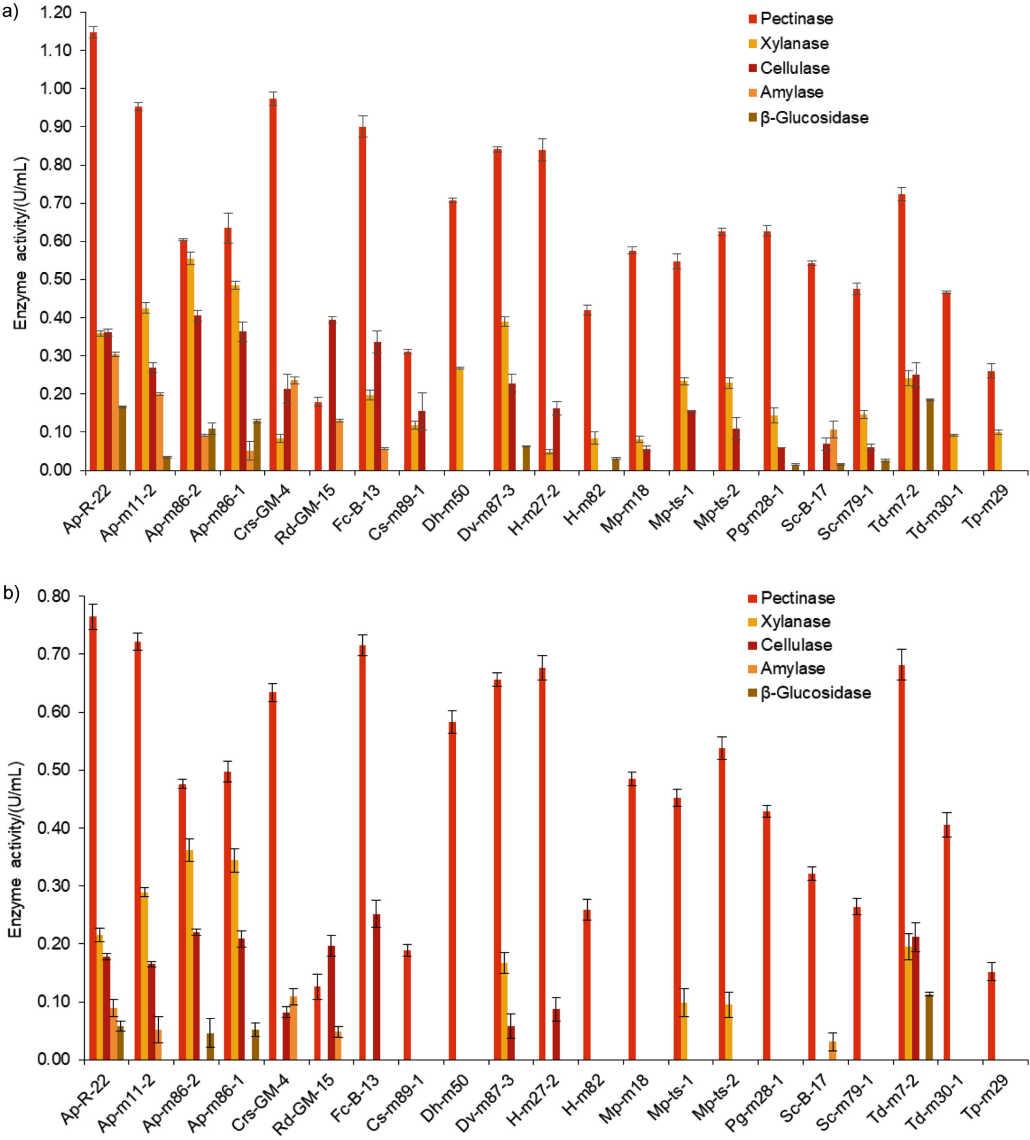
Enzyme activities of oenological importance at: a) 28 °C and b) 15 °C of selected strains (mean value±S.D., *N*=3). One unit of enzyme activity (U) was defined as the amount of enzyme required to release 1 μmol of reducing sugar per min at pH=3.8

Fig. 1 shows the different activities assayed in the enzyme extracts and produced by the strains under study at two temperatures, 15 and 28 °C. Pectinolytic activity was the main observed enzyme activity, reaching significant levels compared with the other two assayed activities. This activity was particularly predominant at low temperature and was the only activity detected in several of the studied strains, with the exception of *A. pullulans* and *T. delbrueckii*, which demonstrated a very broad enzyme profile.

As observed in previous studies, *A. pullulans* is the most abundant species on the grape surface, in fresh grape juice and during very early fermentation stages (*4*, *19*, *39*, *40*). This species has been reported to produce a great diversity of extracellular enzymes such as pectinases, cellulases, xylanases, glycosidases and proteases (*1*, *3*, *19*). In fact, in the present study it was the only species that showed an enzyme pool containing all five aforementioned activities, confirming the presence of a very extensive polysaccharidase enzyme system, at least consisting of the assayed activities (pectinase, xylanase, cellulase and amylase) as well as other complementary activities such as proteases and β-glucosidases. Within this species, the Ap-R-22 and Td-m11-2 strains were remarkable, as they showed the highest values of pectinolytic activity at both assay temperatures (1.149 and 0.954 U/mL at 28 °C, and 0.765 and 0.722 U/mL at 15 °C, respectively). *A. pullulans* is a yeast-like euascomycete fungus, meaning that despite having yeast phases of growth, it is multicellular and, as such, an excellent producer of lytic enzymes. Given the trophic and ecological roles of these fungi, degradation of organic matter, they release enzymes for primary degradation to the environment, and during later stages they provide yeasts responsible for completing the degradation. Based on the present results and according to a previous report (*3*), *A. pullulans* demonstrated the best activity profiles of oenological importance. This yeast-like fungus is very common in the phyllosphere and carposphere of fruit and vegetable crops and has a potential action against phytopathogenic fungi (*41*). According to Onetto *et al.* (*42*), the genus *Aureobasidium* is a central component of the microbial community of grape must, and their studies revealed the potential of the species to affect the composition of grape must through the production of polymers and extracellular enzymes, as well as through modulation of the fermentation kinetics by competition for trace elements.

Continuing with the screening of carbohydrolase-secreting microorganisms, and in agreement with Merín and Morata de Ambrosini (*3*), basidiomycetes were the second most abundant isolates, especially those belonging to the genera *Cryptococcus* and *Rhodotorula*. Only *C. saitoi* produced enzymes with increased activity and a complete enzyme pool considering the assayed activities. Strain *C. saitoi* Crs-GM-4 reached a pectinolytic activity of 0.634 U/mL at 15 °C and 0.974 U/mL at 28 °C, while *R. dairenensis* Rd-GM-15 showed a different enzyme composition: no xylanase or β-glucosidase activity, little pectinase activity and cellulase as the main carbohydrase activity at 15 and 28 °C (0.197 and 0.394 U/mL, respectively). *F. capsuligenum* Fc-B-13 produced a polysaccharidase enzyme pool with high pectinolytic activity (0.715 U/mL at 15 °C and 0.901 U/mL at 28 °C). Previously, Merín *et al.* (*20*) studied *F. capsuligenum* strains and they found good pectinase activity at 12 and 28 °C (0.77 and 1.15 U/mL, respectively). Their report mentioned for the first time the ability of this species to produce pectinases.

Subsequently, the oxidative and weakly fermentative hemiascomycete yeasts belonging to the genera *Candida*, *Debaromyces*, *Hanseniaspora*, *Metschnikowia* and *Pichia* had a less complete enzyme pool, with lower levels of enzyme activities. Previously, production of polysaccharidase activities such as pectinases, cellulases, amylases and proteases in other fruits or fermentation processes by the genera *Debaryomyces* (*43*), *Metschnikowia* (*1*), *Hanseniaspora*, *Candida* and *Pichia* (*44*) had been reported. The findings for strain *D. vanrijiae* Dv-m87-3 were particularly interesting, as it showed significant levels of pectinase (0.656 and 0.841 U/mL at 15 and 28 °C, respectively), xylanase (0.167 and 0.390 U/mL at 15 and 28 °C, respectively), cellulase (0.058 and 0.228 U/mL at 15 and 28 °C, respectively) and β-glucosidase (0.062 U/mL at 28 °C) activities, with a spectrum similar to that of basidiomycete yeasts. There are reports on pectinolytic activity by *Debaromyces hansenii* and *Debaromyces polymorphus* strains present in tropical fruits, whereas xylanolytic activity was only observed in *D. hansenii* and *D. vanrijiae* strains from oenological origin (*45*). Cellulolytic activity is not abundant in wine yeasts, although this activity has been reported in *D. hansenii* isolated from decayed wood (*46*).

Likewise, the apiculated yeast *H. uvarum*/*K. apiculata* seems to be the most common grape berry species worldwide, which is consistent with its predominance at the beginning of spontaneous must fermentations (*38*). It should be mentioned that *Hanseniaspora* sp. H-m27-2 had a particular enzyme profile, almost exclusively consisting of pectinolytic activity, and reached significantly high levels (0.677 and 0.840 U/mL at 15 and 28 °C, respectively).

Finally, two strongly fermentative hemiascomycete yeasts, *S. cerevisiae* and *T. delbrueckii*, were also observed in the present isolation and selection study. These strains produce lytic enzymes, which are of great interest because they are able to produce plant cell wall-degrading enzymes, while carrying out alcohol fermentation. However, these results differ from a previous study by Merín *et al.* (*4*), who did not detect pectinolytic activity among ascomycetous yeasts isolated from grapes and during fermentation.

The species *S. cerevisiae* is rarely isolated from grapes using traditional sampling techniques (*47*). Most *S. cerevisiae* strains normally used in winemaking do not show the ability to degrade pectin substrates. Only a few wild strains have been reported to possess the ability to degrade pectin during wine fermentation (*48*). In the present study, two *S. cerevisiae* strains, one isolated from the grape surface (Sc-m79-1) and one from winery equipment (Sc-B-17) (*20*), were able to secrete pectinases, reaching enzyme activities of 0.264 and 0.321 U/mL at 15 °C, and 0.476 and 0.543 U/mL at 28 °C, respectively. These findings could be related to the fact that some grapes used for isolation had an advanced state of maturity.

Regarding *T. delbrueckii*, strain Td-m7-2 showed significantly higher pectinolytic activity at both assay temperatures (0.682 and 0.723 U/mL at 15 and 28 °C, respectively), and xylanase (0.195 and 0.241 U/mL), cellulase (0.212 and 0.250 U/mL) and β-glucosidase (0.113 and 0.185 U/mL) activities were also excellent. This species is characterized by its decent oenological characteristics such as low acetic acid production, higher glycerol concentration and a better composition of volatile aromatic compounds in wines (*49*). Recent reports indicate its ability to promote malolactic fermentation in wines with a high polyphenol content (*50*). However, the ability of this species to produce extracellular enzymes has been studied little (*2*, *51*). *T. delbrueckii* Td-m30-1 showed a different behaviour, as it only presented pectinase activity, but less than strain Td-m7-2, and it was negative for the other determined activities, suggesting that production of polysaccharidases is strain-dependent.

On the other hand, amylase production was observed in all basidiomycetes and all *A. pullulans* strains assayed, while it was negative in hemiascomycetes, with the exception of *S. cerevisiae* Sc-B-17.

### Technological effects of extracellular enzyme extracts on short macerations of grape must

Table 3 shows the effects of microbial enzyme extracts on different technological parameters evaluated after short macerations of Malbec must at 15 and 28 °C. Musts treated with enzymes showed differences in the extraction of total polyphenols during the maceration. All enzymatically treated musts had total polyphenol index (TPI) values that were higher than that of the control treatment without enzymes (C1) at both assay temperatures. *A. pullulans* Ap-m11-2 had the highest TPI values (59.1±0.4 and 58.6±0.0 at 15 and 28 °C, respectively). However, the other *A. pullulans* extracts had similar values, which were the highest in macerations carried out at 15 °C. Similarly, the *T. delbrueckii* Td-m7-2 extract produced a TPI value during maceration which was higher at 15 than at 28 °C, and also higher than with the three *Metschnikowia pulcherrima* extracts. The remaining extracts produced slightly lower TPI values, probably as a result of the effect of lower molecular mobility due to the low temperature. Our results are in agreement with those observed by Belda *et al.* (*1*) for the same parameter. In particular, this study indicates the potential of *M. pulcherrima* in co-culture with *S. cerevisiae* on a semi-industrial scale to improve the colour properties in red wine owing to its pectinolytic activities at 12 and 28 °C. Likewise, the TPC was the highest in the must treated with Ap-m11-2 extract at both maceration temperatures (750.5±0.6 and 746.8±1.2 at 15 and 28 °C, respectively). Moreover, the TPC in all *M. pulcherrima* extracts and in those of *A. pullulans* Ap-R-22, *T. delbrueckii* Td-m7-2, *D. vanrijiae* Dv-m87-3 and *R. dairenensis* Rd-GM-15 was significantly higher at 15 than at 28 °C.

**Table 3:** Effect of microbial enzyme extracts on the extraction of pigments, total polyphenol content (TPC) and antioxidant activity (AA) of Malbec grape must macerations at 28 and 15 °C

Assay	TPI		CI		Hue		L*		a*		b*		Δ*E*		*γ*(GAE)/(mg/L)				
*t*=28 °C	TPC		AA		
C1	47.5±0.1		9.70±0.10		0.760±0.002		73.2±1.6		26.9±1.0		4.1±1.5		-		624.1±1.0			420.2±2.1	
C2	53.6±0.2		10.50±0.04		0.702±0.004		70.3±1.5		30.2±1.4		1.2±1.3		5.3		719.1±1.4			473.8±1.4	
Ap-R-22	(55.4±0.0)^ab^		(11.71±0.03)^ab^		(0.671±0.003)^ab^		(66.8±1.1)^ab^		(32.8±1.2)^a^		(1.0±1.2)^a^		9.2		(741.2±0.7)^ab^			(527.2±1.3)^ab^	
Ap-m11-2	(58.6±0.0)^ab^		(12.61±0.05)^ab^		(0.572±0.001)^ab^		(64.5±1.3)^ab^		(30.7±1.0)^a^		(0.5±1.6)^a^		10.2		(746.8±1.2)^ab^			(526.9±1.1)^ab^	
Ap-m86-2	(56.6±0.2)^ab^		(11.53±0.04)^ab^		(0.643±0.002)^ab^		(67.2±1.4)^ab^		(31.5±0.9)^a^		(1.8±0.8)^a^		7.9		(744.1±1.4)^ab^			(516.1±0.8)^ab^	
Ap-m86-1	(55.6±0.4)^ab^		(10.91±0.02)^ab^		(0.670±0.003)^ab^		(66.4±1.2)^ab^		(30.8±0.9)^a^		(2.0±1.4)^a^		8.1		(742.7±1.1)^ab^			(511.6±2.3)^ab^	
Crs-GM-4	(54.2±0.2)^ab^		(11.50±0.02)^ab^		(0.650±0.003)^ab^		(67.1±1.5)^ab^		(28.9±1.5)^a^		(2.9±1.3)		6.5		(722.3±0.6)^a^			(484.2±1.3)^ab^	
Rd-GM-15	(49.1±0.1)^ab^		(11.20±0.02)^ab^		(0.750±0.004)^b^		(68.3±0.9)^a^		(31.0±2.3)^a^		(1.9±1.4)^a^		6.8		(670.9±0.5)^ab^			(478.6±1.9)^a^	
Fc-B-13	(56.2±0.2)^ab^		(11.10±0.03)^ab^		(0.673±0.001)^ab^		(66.3±1.2)^ab^		(30.2±1.3)^a^		(2.7±1.8)^a^		7.8		(728.2±1.7)^ab^			(489.0±0.9)^ab^	
Cs-m89-1	(47.7±0.5)^b^		(9.90±0.02)^ab^		(0.773±0.004)^b^		(68.9±1.0)^a^		(27.1±1.1)		(3.2±1.1)^b^		4.4		(633.4±1.1)^ab^			(472.6±1.7)^a^	
Dh-m50	(51.2±0.2)^ab^		(10.70±0.01)^ab^		(0.641±0.004)^ab^		(68.5±1.0)^ab^		(30.5±2.1)^a^		(2.1±1.0)^a^		6.2		(689.5±0.6)^ab^			(480.6±1.0)^a^	
Dv-m87-3	(54.6±0.4)^a^		(11.81±0.01)^ab^		(0.670±0.006)^ab^		(65.9±1.4)^ab^		(30.8±1.3)^a^		(2.8±1.1)		8.4		(718.9±0.7)^a^			(484.7±1.1)^ab^	
H-m27-2	(55.1±0.3)^ab^		(11.55±0.01)^ab^		(0.730±0.006)^ab^		(68.1±1.3)^ab^		(29.5±2.0)		(3.4±1.7)^b^		5.8		(729.3±0.4)^ab^			(485.4±1.7)^ab^	
H-m82	(48.0±0.3)^b^		(10.42±0.04)^b^		(0.750±0.004)^b^		(69.5±0.9)^a^		(27.8±1.4)		(3.5±1.3)^b^		3.8		(646.6±1.0)^ab^			(478.3±0.7)^a^	
Mp-m18	(51.9±0.1)^ab^		(10.90±0.02)^ab^		(0.672±0.005)^ab^		(67.0±1.8)^ab^		(30.8±1.8)		(2.3±1.2)^a^		7.5		(706.3±1.1)^ab^			(480.3±0.8)^a^	
Mp-Ts-1	(53.1±0.1)^a^		(10.80±0.03)^ab^		(0.640±0.002)^ab^		(66.2±1.9)^ab^		(28.9±1.1)		(2.0±0.9)^a^		7.6		(710.8±1.0)^ab^			(481.9±0.6)^ab^	
Mp-Ts-2	(54.4±0.1)^ab^		(11.50±0.04)^ab^		(0.611±0.003)^ab^		(65.3±1.7)^ab^		(31.3±1.0)		(1.6±1.7)^a^		9.4		(715.0±0.8)^a^			(484.7±1.0)^ab^	
Pg-m28-1	(53.4±0.2)^b^		(10.81±0.02)^ab^		(0.790±0.003)^ab^		(71.1±0.8)		(27.4±1.9)		(3.1±1.1)^b^		2.4		(711.7±0.6)^a^			(477.5±1.8)^a^	
Sc-B-17	(53.5±0.4)^a^		(11.13±0.04)^ab^		(0.721±0.005)^a^		(68.8±0.9)^a^		(30.5±1.9)		(3.1±1.2)^b^		5.8		(717.3±0.1)^a^			(483.1±2.1)^ab^	
Sc-m79-1	(51.0±0.4)^ab^		(11.10±0.02)^ab^		(0.660±0.003)^ab^		(67.2 ± 1.9)^ab^		(29.8±1.3)		(2.5±1.4)^a^		6.8		714.1 ± 0.5^*1^			(482.1±2.3)^ab^	
Td-m7-2	(57.9±0.1)^ab^		(11.90±0.03)^ab^		(0.631±0.002)^ab^		(66.2±1.1)^ab^		(31.9±1.5)^a^		(0.9±1.1)^a^		9.2		(735.5±0.6)^ab^			(508.3±1.4)^ab^	
Td-m30	(48.4±0.3)^b^		(10.70±0.01)^ab^		(0.735±0.003)^ab^		(69.3±1.6)^a^		(28.1±2.1)		(3.2±1.7)^b^		4.2		(649.4±1.0)^ab^			(475.5±1.9)^a^	
Tp-m29	(48.0±0.5)^b^		(10.61±0.02)^a^		(0.724±0.003)^ab^		(71.4±1.8)		(27.8±1.4)		(2.9±1.3)		2.3		(644.3±1.0)^ab^			(471.2±1.1)^a^	
*t* =15 °C																				
C1	47.7±0.2		9.80±0.01		0.760±0.005		74.1±1.4		26.2±1.2		3.8±1.0		-		628.7±1.2			440.6±2.2	
C2	54.1±0.1		10.90±0.03		0.612±0.005		70.6±1.1		31.0±1.5		-1.0±1.1		7.6		724.8±0.5			482.9±1.9	
Ap-R-22	(56.6±0.1)^ab^		(12.10±0.03)^ab^		(0.610±0.003)^a^		(67.2±1.0)^ab^		(34.0±1.1)^ab^		(-1.2±0.8)^a^		11.5		(749.1±0.5)^ab^			(553.3±1.9)^ab^	
Ap-m11-2	(59.1±0.4)^ab^		(12.71±0.03)^ab^		(0.553±0.003)^ab^		(65.0±1.1)^ab^		(35.1±1.0)^ab^		(-0.5±0.9)^a^		13.4		(750.5±0.6)^ab^			(548.0±1.4)^ab^	
Ap-m86-2	(57.1±0.1)^ab^		(11.55±0.08)^ab^		(0.621±0.008)^a^		(67.9±1.7)^ab^		(32.3±1.4)^a^		(2.0±1.1)		8.9		(747.2±0.3)^ab^			(537.6±0.9)^ab^	
Ap-m86-1	(55.9±0.3)^ab^		(10.72±0.01)^ab^		(0.550±0.002)^ab^		(68.0±0.9)^a^		(31.9±1.7)^a^		(2.7±1.2)		8.4		(747.0±0.7)^ab^			(531.9±1.5)^ab^	
Crs-GM-4	(53.9±0.1)^a^		(11.50±0.03)^ab^		(0.581±0.001)^ab^		(69.4±1.4)^a^		(29.9±1.4)^a^		(2.1±0.9)		6.2		(720.2±0.9)^ab^			(496.9±1.3)^ab^	
Rd-GM-15	(50.8±0.1)^ab^		(11.20±0.01)^ab^		(0.650±0.002)^ab^		(70.4±1.4)^a^		(27.8±0.9)		(2.3±1.4)		4.3		(675.3±0.4)^ab^			(490.3±1.2)^a^	
Fc-B-13	(56.2±0.2)^ab^		(11.40±0.03)^ab^		(0.610±0.006)^a^		(69.7±1.4)^a^		(27.1±1.0)^b^		(3.7±1.1)^b^		4.5		(726.3±0.7)^a^			(506.1±1.7)^ab^	
Cs-m89-1	(48.7±0.2)^b^		(10.30±0.06)^ab^		(0.620±0.002)^a^		(71.2±2.1)		(26.5±1.2)^b^		(3.4±0.8)^b^		2.9		(631.3±1.0)^b^			(480.2±0.8)^a^	
Dh-m50	(52.8±0.2)^ab^		(10.73±0.01)^ab^		(0.572±0.002)^ab^		(71.1±1.6)		(28.2±0.9)		(3.4±0.8)^b^		3.6		(688.5±0.1)^ab^			(489.9±1.8)^a^	
Dv-m87-3	(55.2±0.1)^ab^		(11.10±0.04)^ab^		(0.570±0.004)^ab^		(68.6±1.1)^a^		(30.1±0.8)^a^		(3.0±1.3)^b^		6.8		(721.9±0.7)^a^			(498.4±0.9)^ab^	
H-m27-2	(52.5±0.2)^ab^		(11.43±0.04)^ab^		(0.680±0.001)^ab^		(72.0±0.9)		(28.0±1.2)		(2.8±0.9)^b^		2.9		(724.5±1.3)^a^			(499.2±2.1)^ab^	
H-m82	(48.9±0.2)^b^		(10.32±0.03)^ab^		(0.634±0.001)^a^		(72.8±1.7)		(28.2±1.7)		(3.3±0.9)^b^		2.4		(642.9±0.9)^ab^			(488.1±0.7)^a^	
Mp-m18	(52.6±0.1)^ab^		(10.10±0.08)^a^		(0.610±0.004)^a^		(69.9±1.1)^a^		(31.3±1.2)^a^		(2.2±0.8)		6.8		(711.3±1.8)^ab^			(493.5±1.4)^ab^	
Mp-Ts-1	(54.4±0.1)^a^		(11.10±0.03)^ab^		(0.635±0.001)^ab^		(69.5±1.4)^a^		(30.2±1.6)^a^		(2.5±1.2)		6.2		(714.0±0.6)^ab^			(505.3±0.8)^ab^	
Mp-Ts-2	(55.1±0.2)^ab^		(11.81±0.05)^ab^		(0.624±0.002)^a^		(68.1±1.6)^a^		(32.0±0.9)^a^		(1.7±1.5)		8.6		(720.0±0.9)^ab^			(514.1±1.0)^ab^	
Pg-m28-1	(51.3±0.2)^ab^		(10.51±0.06)^ab^		(0.690±0.008)^ab^		(73.2±1.7)^b^		(25.8±1.8)^b^		(3.9±0.9)^b^		1.0		(707.6±0.7)^ab^			(488.4±2.4)^a^	
Sc-B-17	(51.6±0.2)^ab^		(11.10±0.03)^ab^		(0.660±0.004)^ab^		(71.0±1.9)		(29.2±1.4)^b^		(4.1±1.1)^a^		4.3		(713.6±0.1)^ab^			(494.9±1.4)^ab^	
Sc-m79-1	(51.0±0.4)^ab^		(11.20±0.03)^ab^		(0.631±0.007)^ab^		(70.7±1.5)^a^		(30.2±1.7)^a^		(1.7±0.6)		5.7		(709.1±1.1)^ab^			(494.0±0.6)^ab^	
Td-m7-2	(58.5±0.1)^ab^		(11.94±0.02)^ab^		(0.610±0.001)^ab^		(67.3±1.0)^ab^		(32.5±1.7)^a^		(1.0±1.0)^a^		9.7		(739.5±0.6)^ab^			(528.3±1.3)^ab^	
Td-m30	(48.1±0.2)^b^		(11.01±0.02)^ab^		(0.660±0.002)^ab^		(72.5±1.3)		(26.6±1.2)^b^		(3.5±1.3)^b^		1.7		(648.3±0.6)^ab^			(484.4±0.4)^a^	
Tp-m29	(48.2±0.3)^b^		(10.20±0.03)^ab^		(0.690±0.001)^ab^		(72.1±1.1)		(26.8±1.5)^b^		(3.3±1.2)^b^		2.2		(643.5±0.2)^ab^			(478.7±1.6)^a^	
TPI=total polyphenol index, CI=colour intensity, GAE=gallic acid equivalents, Ap=*Aureobasidium pullulans*, Crs=*Cryptococcus saitoi*, Rd=*Rhodotorula dairenensis*, Fc=*Filabasidium capsuligenum*, Cs=*Candida stellata,* Dh=*Debaryomyces hansenii,* Dv*=Debaryomyces vanrijiae,* H=*Hanseniaspora* sp*.,* Mp=*Metschnikowia pulcherrima,* Pg*=Pichia guilliermondii,* Sc*=Saccharomyces cerevisiae,* Td*=Torulaspora delbrueckii,* Tp*=Torulaspora pretoriensis*, C1=control without enzymes, C2=control with commercial enzyme (1 U/mL). ^ab^significant differences with respect to both controls C1 and C2, ^a^significant differences with respect to the control without enzyme C1, ^b^significant differences with respect to the control with enzyme treatment C2. Without letters, there are no significant differences with respect to the controls																			

Quantification of the antioxidant activity (AA) by means of DPPH˙ radical scavenging is based on the capacity of the sample to reduce free radicals, which is proportional to the antioxidant content. In our case, the AA values of all enzymatically treated musts were higher at 15 than at 28 °C. All *A. pullulans* enzyme extracts had a high antioxidant capacity, particularly Ap-R-22 and Ap-m11-2, which showed a significantly higher activity at 15 °C than the other strains (553.3 and 548.0 mg/L, respectively). Other prominent enzyme extracts regarding their high antioxidant activity at low temperatures were (in mg/L): Td-m7-2 (528.3), Fc-B-13 (506.1) and the two *M. pulcherrima* extracts: Mp-ts-2 and Mp-ts-1 (514.1 and 505.3, respectively).

Therefore, macerations with highest TPI, TPC and antioxidant capacity values corresponded to the most extensive multi-enzyme systems. The antioxidant capacity is related to the content of anthocyanins and total polyphenols. Each phenolic compound contributes proportionately and differently to this activity. Di Carlo *et al*. (*52*) observed a strong linear correlation between the antioxidant activity and TPI values.

The colour of a sample is defined by the colour intensity (CI) and the hue. The latter parameter represents the relative importance of yellow over red. According to the results shown in Table 3, a significant decrease in the hue was observed after all macerations with enzymes, even with the commercial enzyme (C2), compared to the control (C1). There were no statistically significant differences in CI between Ap-R-22 and Ap-m11-2 extracts, but there were in the hue, which would indicate that the Ap-m11-2 sample extracted more red pigments. Ap-m86-1 had a lower CI than the other extracts at 15 and 28 °C, but the hue was also lower. In the case of Crs-GM-4, there were no significant differences in the CI between both temperatures, but the hue was significantly lower at 15 than at 28 °C. This is a favourable effect of the low temperature on the colour composition. Additionally, the microbial extracts with the most extensive enzyme systems produced the highest values of the CIELAB parameters and highest Δ*E*.

Filterability and clarification of the macerated musts were also assessed at 15 and 28 °C (Table 4). At both temperatures and for all assayed enzyme extracts, filtration time was shorter than that of control without the enzyme. Furthermore, for all enzyme extracts, the filtration time at 15 °C was shorter than that recorded at 28 °C. In particular, macerations with Td-m7-2 and Ap-R-22 extracts required the shortest filtration times at 15 °C (249.2 and 250.4 s/mL, respectively), followed by those with Ap-m11-2 and Dv-m87-3 (256.3 and 255.0 s/mL, respectively). Regarding clarification, the formation of pectin flocs facilitates the production of a clear supernatant through elimination of the colloidal particles of the must. Hence, musts treated with Td-m7-2 and Ap-m86-2 were the most efficient, with an increase in transmittance of more than 4 times at both maceration temperatures. Other extracts with good clarification performance at low temperature were those of *D. vanrijiae* Dv-m87-3, *A. pullulans* Ap-R-22 and *Hanseniaspora* sp. H-m27-2. These results demonstrate that filtration and clarification are positively correlated with strains with very wide hydrolytic enzyme activity profiles for the degradation of plant cell wall polymers.

**Table 4:** Effect of microbial enzyme extracts on filterability and clarification of white grape must during macerations at 28 and 15 °C

Assay	Temperature/°C			
	28	15	28	15
	Filterability/(s/mL)		*T*_650 nm_/%	
C1	388.0±0.4	380.1±0.1	12.9±1.2	12.8±1.2
C2	238.0±0.4	210.4±0.2	65.0±1.3	67.5±1.3
Ap-R-22	(266.6±1.1)^ab^	(250.4±1.0)^ab^	(48.9±1.1)^ab^	(55.4±1.0)^ab^
Ap-m11-2	(268.5±0.9)^ab^	(256.4±1.0)^ab^	(40.8±1.3)^ab^	(42.5±0.8)^ab^
Ap-m86-2	(354.0±1.4)^ab^	(283.2±1.0)^ab^	(53.0±1.4)^ab^	(55.2±1.2)^ab^
Ap-m86-1	(312.3±0.8)^ab^	(296.6±0.8)^ab^	(46.7±1.4)^ab^	(47.9±1.3)^ab^
Crs-GM-4	(378.0±1.4)^b^	(361.0±1.1)^ab^	(42.6±1.8)^ab^	(48.9±0.6)^ab^
Rd-GM-15	(315.7±1.6)^ab^	(282.5±0.8)^ab^	(45.4±1.6)^ab^	(46.4±1.0)^ab^
Fc-B-13	(378.3±0.9)^ab^	(355.0±1.4)^ab^	(40.5±1.8)^ab^	(45.0±1.0)^ab^
Cs-m89-1	(370.5±2.1)^ab^	(351.9±0.9)^ab^	(31.7±1.2)^ab^	(37.5±0.7)^ab^
Dh-m50	(365.5±0.6)^ab^	(327.8±1.3)^ab^	(33.4±1.5)^ab^	(35.3±1.0)^ab^
Dv-m87-3	(272.5±0.7)^ab^	(255.0±1.2)^ab^	(50.5±1.3)^ab^	(54.4±0.9)^ab^
H-m27-2	(299.0±1.5)^ab^	(264.3±1.4)^ab^	(48.9±1.3)^ab^	(53.4±0.7)^ab^
H-m82	(363.0±1.5)^ab^	(346.5±0.9)^ab^	(29.7±1.4)^ab^	(32.3±0.9)^ab^
Mp-m18	(379.9±0.8)^b^	(368.2±0.9)^ab^	(34.4±1.3)^ab^	(43.1±1.1)^ab^
Mp-Ts-1	(375.5±0.8)^ab^	(322.5±1.5)^ab^	(33.5±1.0)^ab^	(34.3±0.8)^ab^
Mp-Ts-2	(340.3±0.9)^ab^	(336.6±0.8)^ab^	(43.5±1.7)^ab^	(43.0±1.0)^ab^
Pg-m28-1	(390.0±1.4)^b^	(379.6±0.9)^b^	(30.7±1.1)^ab^	(33.0±1.5)^ab^
Sc-B-17	(324.3±0.9)^ab^	(309.2±1.3)^ab^	(39.4±1.6)^ab^	(46.5±0.9)^ab^
Sc-m79-1	(368.0±1.5)^ab^	(335.6±0.8)^ab^	(33.4±0.7)^ab^	(36.0±1.1)^ab^
Td-m7-2	(271.1±1.3)^ab^	(249.3±1.1)^ab^	(51.6±2.0)^a^	(57.0±1.5)^a^
Td-m30	(383.7±0.9)^b^	(370.5±0.9)^b^	(36.4±1.5)^ab^	(38.2±1.0)^ab^
Tp-m29	(380.7±1.1)^b^	(366.2±1.4)^ab^	(26.1±1.7)^ab^	(33.2±1.1)^ab^
C1=control without enzymes, C2=control with commercial enzyme (1 U/mL). ^ab^significant differences with respect to both controls C1 and C2, ^a^significant differences with respect to the control without enzyme treatment C1, ^b^significant differences with respect to the control with enzyme treatment C2. Without letters, there are no significant differences with respect to the controls				

### Principal component and cluster analyses

**Fig. 2:**
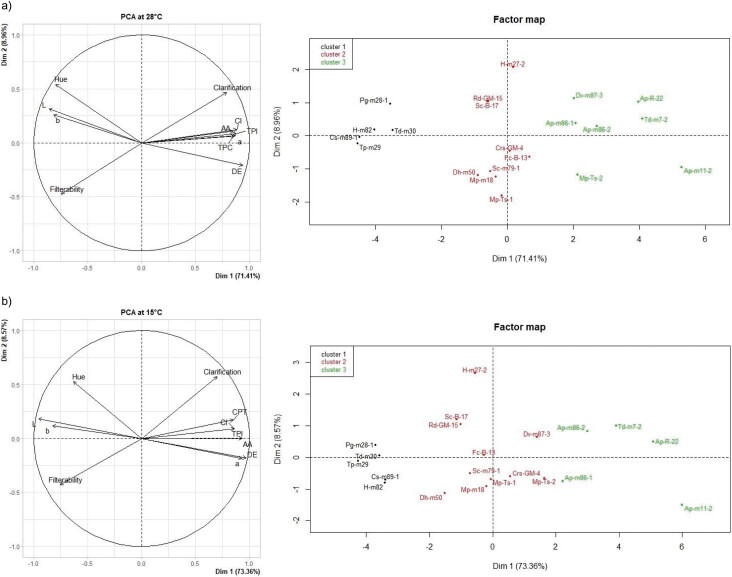
Biplot graphs of the first two principal components using PCA and Factor Map for the effects of microbial enzyme extracts on technological parameters assayed for short maceration of Malbec must at: a) 28 °C and b) 15 °C

In order to select strains with multiple enzyme activities of great oenological importance based on the effects observed with macerations carried out with Malbec must, principal component analysis (PCA) was applied. At 28 °C (Fig. 2a), the first two dimensions explain 80.4% of the variability, with component one (PC1) representing 71.4% and component two (PC2) 9.0%. At 15 °C, PC1 represents 73.4% and PC2 8.6% of the variability (Fig. 2b). At both temperatures, PC1 positively correlated with the following parameters: clarification, TPI, TPC, CI, a*, Δ*E* and AA. All *A. pullulans* enzyme extracts together with Td-m7-2, Dv-m87-3 and Mp-ts-2 showed a similar behaviour regarding their relationship with the variables mentioned in component one. In turn, enzyme extracts presenting negative values for PC1 show an inverse correlation with these parameters. In this case, they correspond to the strains with low enzyme activity and with enzyme systems comprising only one or two of the assayed activities. Therefore, extracts that have multiple enzymes with a broad range of activities are represented by positive PC1 values, while those that show lower levels and types of enzyme activity are negative, and the intersection of the components is occupied by extracts with intermediate enzyme activities.

PC2 was represented with significant weight by the following parameters: filterability, hue, clarity (L*) and component b*. The hue, L* and b* are variables that are negatively related to the colour of red wine, and hence Pg-m28-1, H-m82, Cs-m89-1, Tp-m29 and Td-m30 extracts had a negative impact on the colour of the must during maceration. Filterability in the negative quadrant indicates a low efficiency of this parameter, resulting in longer filtration times.

**Fig. 3:**
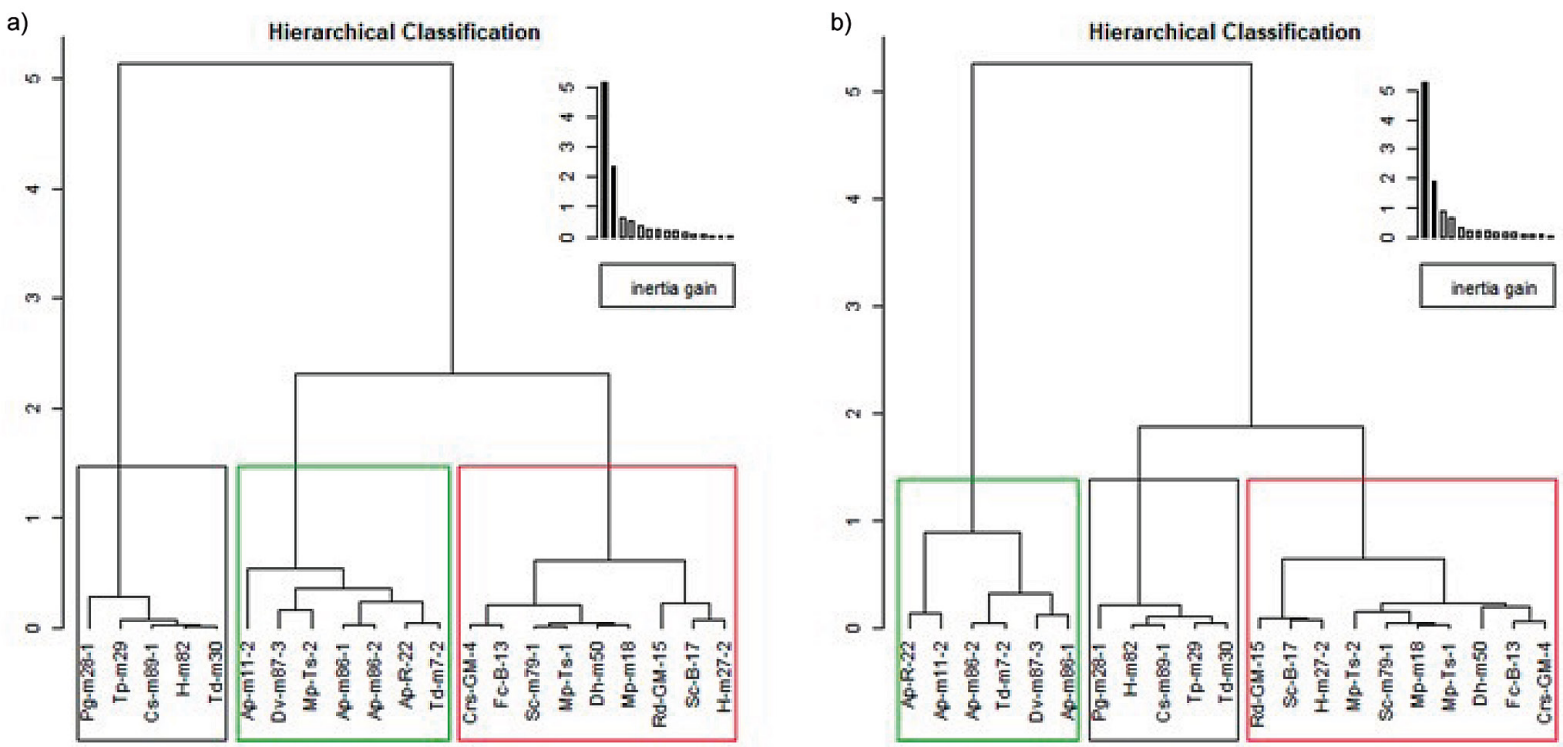
Cluster analysis of microbial enzyme extracts after their technological effects on maceration of Malbec grape must at: a) 28 °C and b) 15 °C

Cluster analysis (Fig. 3) demonstrates that all *A. pullulans* extracts (Ap-R-22, Ap-m11-2, Ap-m86-1 and Ap-m86-2) as well as those of *T. delbrueckii* Td-m7-2, *D. vanrijiae* Dv-m87-3 and *M. pulcherrima* Mp-ts-2 generated the same cluster at 28 °C. At 15 °C, however, only the Ap-R-22, Ap-m11-2, Ap-m86-2 and Td-m7-2 extracts generated a cluster which was different from all the others, showing the ability to improve technological parameters assessed at the two assay temperatures. Consequently, these strains would produce the best blend of enzymes to improve the chromatic characteristics, clarification and filterability as well as the extraction of bioactive compounds during maceration of the grape must.

## CONCLUSIONS

The present study assessed the enzymatic potential of epiphytic grape yeasts belonging to 13 species of oenological interest, selected for their ability to produce polysaccharidases. The majority of the assayed strains produced multiple enzymes necessary to degrade a material as complex as the plant cell wall. A great difference in the activity profile could be observed among the yeast species, even within the same species, which indicates the importance of selecting optimal strains for the production of enzymes. All *Aureobasidium pullulans* strains were able to produce carbohydrolases of oenological importance: pectinases, xylanases and cellulases, in addition to amylases, proteases and β-glucosidases. Besides, two strains belonging to the species *Torulaspora delbrueckii* and *Metschnikowia pulcherrima* showed an enzyme spectrum that was very different from the other isolated strains of the same species. Two *Saccharomyces cerevisiae* strains (Sc-m79-1 and Sc-B-17) were effective pectinase producers under oenological conditions, which is an advantage of their use as starter cultures to improve particular wine properties. Strains that produced the most comprehensive enzyme extracts and the highest activity also exhibited the best chromatic and technological properties. The present study shows the enzyme capacity of some non-*Saccharomyces* yeast strains, suggesting their potential use as a co-culture with *S. cerevisiae* to improve the determined sensorial and technological properties, thus enhancing the wine quality. Four *A. pullulans* strains (Ap-R-22, Ap-m11-2, Ap-m86-2, Ap-m86-1) with *T. delbrueckii* m7-2, *Debaryomyces vanrijiae* m87-3 and *M. pulcherrima* ts-2 showed the highest enzyme activities and they were associated with remarkable effects on colour, clarification, filterability and antioxidant activity of the must. The performance of the selected strains and their extracts in winemaking still has to be examined to confirm the technological effects and rule out possible negative effects. Furthermore, these microorganisms can be granted a GRAS status. Hence, besides the use of their enzyme extracts, it is also possible to propose the application of the microorganism itself to constitute a mixed culture able to produce *in situ* maceration enzymes. Therefore, the present study allows expansion of previous findings by our research group, thus contributing to a better understanding of the effect of multiple enzymes, produced by indigenous yeasts and yeast-like organisms on the grape berry and in the must ecosystem, on compound transformations during maceration.
